# Diagnostic Performance and Utility of the Hwabyung Comprehensive Test in Differential Clinical Assessment

**DOI:** 10.3390/diagnostics16081165

**Published:** 2026-04-14

**Authors:** Seok-In Yoon, Hui-Yeong Park, Yerim Jeon, Jiho Pyun, Kieun Lee, Sun-Yong Chung, Jong Woo Kim

**Affiliations:** 1 Department of Neuropsychiatry, College of Korean Medicine, Kyung Hee University, Seoul 02453, Republic of Korea; bort001@naver.com (S.-I.Y.); jadegreen8@naver.com (H.-Y.P.); lovepwr@khu.ac.kr (S.-Y.C.); 2 Department of Neuropsychiatry in Korean Medicine, Kyung Hee University Medicine Center, Seoul 02447, Republic of Korea; yerim770@khu.ac.kr (Y.J.); joep725@khu.ac.kr (J.P.); smhanui18@gmail.com (K.L.); 3 Department of Neuropsychiatry, Kyung Hee University Korean Medicine Hospital at Gangdong, Seoul 05278, Republic of Korea

**Keywords:** Hwabyung, psychosomatic disorders, anger, sensitivity and specificity, ROC curve analysis

## Abstract

**Background:** Hwabyung is a psychosomatic condition characterized by suppressed anger accompanied by somatic distress. Although traditionally considered a culture-bound syndrome, evidence suggests that Hwabyung reflects culturally shaped manifestations of universal emotional and interoceptive processes. The Hwabyung Comprehensive Test (HCT) was developed to assess Hwabyung symptoms; however, its accuracy for clinical identification and differential discrimination requires further validation in clinically representative samples. **Methods:** Patients presenting Hwabyung symptoms were recruited from a university hospital and classified using a structured clinical interview as the reference standard. Participants were categorized into a Hwabyung group (HG; *n* = 100) and a non-Hwabyung group (NHG; *n* = 82), including a non-Hwabyung clinical group (NHCG; *n* = 36) and a non-clinical group (NCG; *n* = 46). The HCT symptom scale, including physical (HCT-P) and emotional (HCT-E) subscales, served as the index test. ROC analyses were conducted to evaluate diagnostic accuracy and optimal cut-off scores. **Results:** For distinguishing HG from NHG, HCT-total demonstrated good diagnostic accuracy for identifying Hwabyung, with an optimal cut-off score of 33.5 (sensitivity = 0.710, specificity = 0.820). In differentiating HG from NHCG, both HCT-total and HCT-P showed fair discriminative performance, with HCT-P exhibiting higher specificity. A cut-off score of 16.5 for HCT-P yielded a sensitivity of 0.540 and a specificity of 0.833. **Conclusions:** The HCT demonstrated good diagnostic accuracy for identifying Hwabyung and fair performance in differentiating it from psychiatric disorders. These findings support a stepwise clinical application in which HCT-total is used for initial screening and HCT-P is a supplementary measure for supporting differential diagnostic decision-making.

## 1. Introduction

Hwabyung is a psychosomatic condition characterized by emotional symptoms such as resentment and anger, along with distinctive physical symptoms including chest tightness and heat sensations [1,2]. Unlike depressive and anxiety disorders, which are primarily defined by affective, cognitive, and behavioral disturbances, Hwabyung is marked by the prominent coexistence of somatic distress. According to traditional East Asian medicine, physical and emotional distress arise from disrupted circulation and imbalance of vital energy (qi, 氣) [3]. From this perspective, Hwabyung is understood as a condition in which suppressed anger leads to upward stagnation of Hwa (火) energy.

Although Hwabyung has traditionally been regarded as a culture-bound syndrome in Korea [4], its underlying mechanism—chronic suppression of anger—may operate across cultures, suggesting that similar symptom patterns can emerge in other cultural or social contexts. Anger is a basic emotion experienced across cultures [5] and involves both emotional and interoceptive processes, including subjective feelings of anger and associated bodily sensations such as chest tightness and localized heat sensations in the chest and fists [6]. Accordingly, Hwabyung can be conceptualized as a culturally patterned expression of these shared emotional and interoceptive mechanisms, rather than a purely culture-specific disorder.

Several diagnostic criteria and assessment tools have been developed for Hwabyung. Kim et al. [2] introduced the Hwa-Byung Diagnostic Interview Schedule, and recent clinical guidelines have provided diagnostic recommendations [7]. Based on these criteria, prevalence estimates in Korea range from 4.2% to 13.3% [8,9,10]. Hwabyung shows high comorbidity with depressive (65%) and anxiety disorders (27%), whereas Hwabyung-only cases account for approximately 22–26% [11,12]. This high comorbidity may reflect shared underlying mechanisms, including chronic negative affect and difficulties in emotion regulation. However, Hwabyung is distinguished by the prominent coexistence of somatic distress and suppressed anger.

Several self-report instruments have also been developed to assess the severity of Hwabyung symptoms. Although the State–Trait Anger Expression Inventory measures anger-related cognitive, affective, and behavioral components, it does not capture the physical symptoms central to Hwabyung. The Hwabyung Scale (HS), developed by Kwon et al. [13], was the first self-report measure designed to assess both physical and emotional symptoms of Hwabyung. Nevertheless, the HS is structured as a single-factor scale without differentiating physical and emotional domains, and it was developed primarily to distinguish Hwabyung from depressive disorders. As a result, its diagnostic sensitivity for Hwabyung itself is limited, and the use of culturally dated expressions (e.g., Han) has raised the need for conceptual and linguistic modernization.

To address these limitations, the Hwabyung Comprehensive Test (HCT) was developed [14]. While the HS reflects a unidimensional structure, the HCT was developed as a multidimensional construct that explicitly distinguishes between physical and emotional symptoms. In addition, the HCT replaces culturally dated terminology such as “Han” with more contemporary and clinically accessible expressions (e.g., resentment-related emotional distress), thereby enhancing interpretability in modern clinical contexts. Furthermore, the HCT extends beyond symptom assessment by incorporating Hwabyung-related stressful life events and personality traits associated with vulnerability to Hwabyung. Previous research has demonstrated that the HCT shows superior classification performance compared with the HS and exhibits strong associations with related emotional variables, including depression, anxiety, and anger, supporting its validity [15].

Despite its contributions, prior validation research on the HCT has several limitations. First, the non-clinical comparison group consisted primarily of university students, limiting its representativeness of non-Hwabyung individuals encountered in clinical settings, resulting in notable age differences between groups. Second, the use of extreme group comparisons between clinically diagnosed Hwabyung patients and asymptomatic student samples may have inflated estimates of sensitivity and specificity, potentially increasing the risk of misclassification when applying the derived cut-off scores in practice. Third, the previous study did not account for the diagnosis of other psychiatric disorders despite the high comorbidity of Hwabyung with depression and anxiety, insufficiently examining the differential diagnostic utility of the HCT.

The present study aims to evaluate the accuracy of the HCT for the clinical identification of Hwabyung and its performance in differentiating Hwabyung from other psychiatric conditions, and to establish clinically applicable cut-off scores. As a follow-up validation study, it addresses the limitations of prior research in several ways. First, by recruiting patients presenting with Hwabyung-related symptoms or distress in a hospital setting, and classifying them based on a structured clinical interview, this study obtained a non-Hwabyung group that more accurately reflects real-world clinical populations. Second, by enrolling help-seeking individuals whose diagnostic status was not determined a priori, and subsequently classifying them after clinical evaluation, this study minimized spectrum bias and derived cut-off scores that are more applicable to routine clinical practice. Third, by simultaneously assessing diagnoses of other psychiatric disorders, this study compared the Hwabyung group with other psychiatric diagnostic groups, and proposed differentiation-oriented cut-off scores, thereby providing practical evidence to support clinical decision-making related to the differentiation of Hwabyung beyond initial screening.

## 2. Materials and Methods

### 2.1. Design

This study was designed as a prospective, single-center, cross-sectional study conducted at a university hospital in Seoul, Republic of Korea. Enrollment was conducted between 14 February 2025 (first participant enrolled) and 27 June 2025 (last participant enrolled). Enrollment was completed during the scheduled study visit following provision of written informed consent and eligibility screening. The study design and reporting followed the STARD guidelines (Supplementary Material S1).

### 2.2. Participants

The eligibility criteria were as follows: participants were included if they (i) reported psychosomatic distress related to Hwabyung, such as anger, feelings of unfairness, chest tightness, or a sensation of internal heat, and (ii) were aged 19 years or older, which corresponds to the legal age of adulthood in the Republic of Korea. Participants were excluded if they (i) exhibited hallucinations (e.g., visual or auditory) or delusions, (ii) experienced organic brain disorders such as dementia, epilepsy, or intellectual or personality disorders, or (iii) had a condition that made it difficult to complete the interviews and assessments conducted in this study (e.g., difficulty in reading, writing, listening, speaking, or comprehension).

The minimum sample size for the receiver operating characteristic (ROC) curve analysis was calculated using MedCalc software version 23.0 [16], and was based on standard methods for estimating the area under the curve (AUC). With a type I error of 0.001, a type II error of 0.005, an AUC of 0.75 (considered a medium level), and a recruitment ratio of 1:1 for positive and negative cases, the required sample size for each group was 80. To account for an anticipated 20% rate of incomplete participation and missing data, the target sample size was set at 100 per group. The type I and type II error levels were set to reduce the risk of false positive conclusions regarding diagnostic performance and to ensure sufficient statistical power to detect a clinically meaningful level of discrimination.

### 2.3. Measurements

#### 2.3.1. Hwabyung Comprehensive Test (HCT)

The HCT [14] is a self-report scale consisting of three components: Hwabyung symptoms (13 items), Hwabyung-related stressful life events (5 items), and personality traits associated with vulnerability to Hwabyung (21 items). In this study, only the HCT symptom scale was used for analysis. The HCT symptom scale consists of two subscales: physical symptoms (HCT-P; 6 items; e.g., “I feel a sense of chest tightness”) and emotional symptoms (HCT-E; 7 items; e.g., “I feel resentment”). All items were rated on a 5-point Likert scale ranging from 0 to 4. Total scores on the HCT ranged from 0 to 52, with subscale scores ranging from 0 to 24 for HCT-P and 0 to 28 for HCT-E. Higher scores indicate greater severity of Hwabyung symptoms. In previous research, the HCT demonstrated good internal consistency, with a Cronbach’s alpha of 0.89 [14]. In this study, Cronbach’s alpha was 0.96 for the HCT, 0.93 for HCT-P, and 0.95 for HCT-E.

#### 2.3.2. Korean Version of the Beck Depression Inventory (BDI)

The BDI [17] is a 21-item self-reporting scale used to assess the severity of depressive symptoms. Each item (e.g., “I feel that the future is hopeless”) was rated on a 4-point Likert scale (0–3), yielding a total score ranging from 0 to 63. Higher scores reflect greater depressive severity. Cronbach’s alpha was 0.85 in a previous study [17] and 0.93 in this study.

#### 2.3.3. Korean Version of the Beck Anxiety Inventory (BAI)

The BAI [18] is a 21-item self-report scale that is used to assess the severity of anxiety symptoms. Each item (e.g., “I am unable to relax”) was rated on a 4-point Likert scale (0–3), yielding a total score ranging from 0 to 63. Higher scores indicate greater anxiety severity. Cronbach’s alpha was 0.91 in previous studies [18] and 0.96 in this study.

### 2.4. Procedure

Participants were recruited using a convenience sampling approach from patients visiting a university hospital in Seoul, Republic of Korea. Individuals who expressed interest after viewing on-site recruitment notices were invited to participate. Interested individuals scheduled a separate study visit, during which, written informed consent was obtained, followed by eligibility screening to determine study inclusion. Recruitment was conducted between 5 February 2025 and 27 June 2025.

Following eligibility screening, participants underwent a structured clinical interview conducted by trained clinicians to diagnose Hwabyung and other psychiatric disorders [2,19]. The interview was conducted as an individual, face-to-face assessment between the clinician and each participant using a structured diagnostic checklist, and included evaluation of core symptoms, functional impairment, and the presence of other medical conditions. Clinicians assessed the presence and clinical significance of symptoms based on predefined diagnostic criteria. This interview served as the reference standard for the diagnosis of Hwabyung given its established reliability and validity [2].

Hwabyung was diagnosed when individuals exhibited both physical and emotional symptoms, reported identifiable stressors associated with these symptoms, and demonstrated impaired psychosocial functioning, with these conditions not attributable to other medical illnesses [2]. Based on the results of the structured clinical interview, participants diagnosed with Hwabyung were classified into the Hwabyung group (HG); those not diagnosed with Hwabyung, but meeting diagnostic criteria for other psychiatric disorders, were classified into the non-Hwabyung clinical group (NHCG); and those not receiving any psychiatric diagnosis were classified into the non-clinical group (NCG).

**Figure 1:**
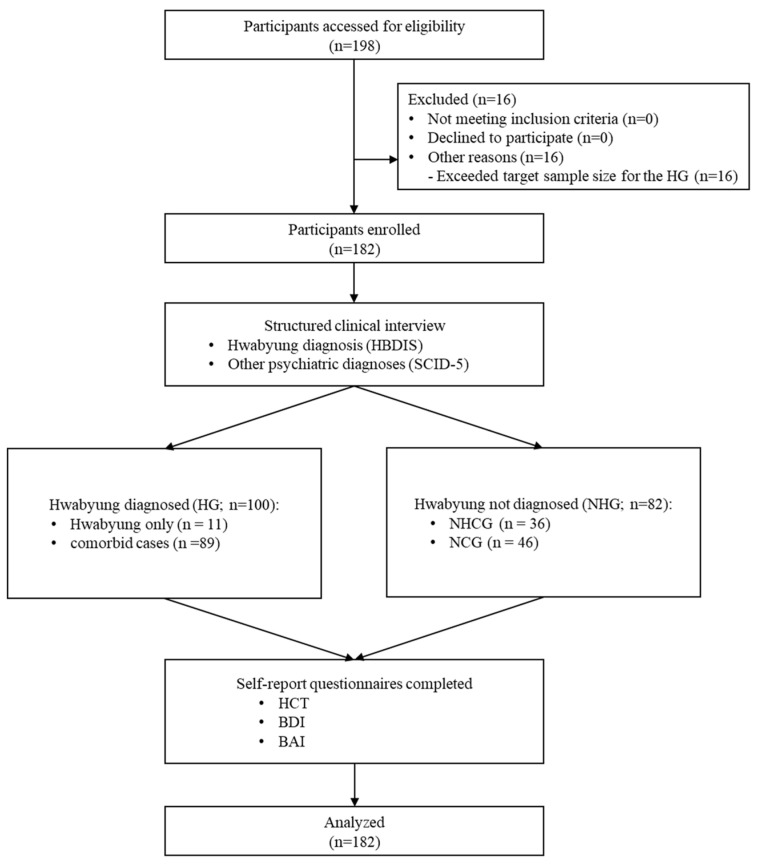
Study flow diagram. BAI = Korean version of Beck Anxiety Inventory; BDI = Korean version of Beck Depression Inventory; HCT = Hwabyung Comprehensive Test, symptom scale (total score); HG = Hwabyung group; NCG = non-clinical group; NHCG = non-Hwabyung clinical group; NHG = non-Hwabyung group.

After the interview, all participants completed a series of self-report questionnaires, including the HCT, BDI, and BAI. The HCT was used as the index test to aid the clinical identification of individuals with Hwabyung. All participants received 50,000 KRW as compensation upon completion of the study. Among the excluded participants (*n* = 16), none were excluded for failure to meet the inclusion criteria. These participants were diagnosed with Hwabyung after the target sample size for the HG (*n* = 100) had already been reached and were therefore excluded for administrative reasons. Recruitment was terminated after the non-Hwabyung group (NHG) reached the minimum required sample size (*n* = 82). Study flow diagram is presented in Figure 1.

### 2.5. Blinding

This study was conducted using a double-blind design. Participants were blinded to their diagnostic classification, determined using the reference standard (structured clinical interview). In addition, the clinicians who conducted the structured interviews were blinded to the results of the index test (HCT), which was completed independently by the participants following the interview. This procedure ensured that the reference standard assessment was not influenced by the index test results.

### 2.6. Statistical Analysis

Statistical analysis was performed using SPSS version 22.0 (IBM Corp., Armonk, NY, USA) and R version 4.1.3. There were no missing data or indeterminate results. Analysis of variance and chi-square tests were used to compare the demographic and clinical characteristics of the participants. Post hoc comparisons were conducted using the Bonferroni method. Statistical significance was set at *p* < 0.05.

Diagnostic accuracy was assessed by calculating sensitivity, specificity, and the AUC. The optimal cut-off score for Hwabyung diagnosis was determined exploratorily using ROC curve analysis to maximize sensitivity and specificity as no pre-specified cut-off value had been established prior to data analysis. 95% confidence intervals (CIs) for the optimal cutoff values were estimated using bootstrap resampling (2000 iterations). Sensitivity and specificity were examined at the lower and upper bounds of the CIs. In addition, positive predictive value (PPV), negative predictive value (NPV), positive likelihood ratio (LR+), and negative likelihood ratio (LR−) were calculated to further evaluate the clinical interpretability of each optimal cut-off score. AUC values of 0.50–0.60 were considered failure, 0.60–0.70 was considered poor, 0.70–0.80 was considered fair, 0.80–0.90 was considered good, and 0.90–1.00 was considered excellent.

Pairwise comparisons of AUC values were conducted using DeLong’s test, and Bonferroni-adjusted *p*-values were examined for the three pairwise comparisons within each classification task.

## 3. Results

### 3.1. Group Comparisons of Demographic and Clinical Characteristics

A total of 100 Hwabyung-positive cases (HG) and 82 Hwabyung-negative cases (non-Hwabyung group (NHG) comprising the non-Hwabyung clinical group (NHCG; *n* = 36) and the non-clinical group (NCG; *n* = 46)) were enrolled. Within the HG, 11 participants were diagnosed exclusively with Hwabyung, whereas 89 participants had at least one comorbid psychiatric disorder. This included major depressive disorder (*n* = 38), recurrent depressive disorder (*n* = 31), panic disorder (*n* = 5), generalized anxiety disorder (*n* = 9), adjustment disorder (*n* = 2), post-traumatic stress disorder (*n* = 17), somatic symptom disorder (*n* = 15), and insomnia disorder (*n* = 61). In the NHCG, psychiatric diagnoses included major depressive disorder (*n* = 13), recurrent depressive disorder (*n* = 12), panic disorder (*n* = 2), generalized anxiety disorder (*n* = 2), adjustment disorder (*n* = 3), post-traumatic stress disorder (*n* = 6), somatic symptom disorder (*n* = 3), and insomnia disorder (*n* = 20). Comorbid diagnoses were not mutually exclusive.

The demographic and clinical characteristics of HG, NHCG, and NCG were compared, and the results are presented in Table 1. There were no significant differences in age among the groups; however, significant group differences were observed for sex and marital status. Post hoc analysis revealed that the proportion of females was significantly higher in the HG than in the NCG (*χ*^2^ = 7.371, *p* = 0.007). In addition, the proportion of married participants was significantly higher in the HG compared with the NCG (*χ*^2^ = 5.964, *p* = 0.015). Significant group differences were also observed in Hwabyung symptoms, Hwabyung-related stressful life events, Hwabyung-related personality traits, depressive and anxiety symptoms. Post hoc analysis indicated that the HG exhibited the highest levels of Hwabyung depression, and anxiety symptoms among the three groups. Moreover, the HG showed significantly higher scores than the NHCG on all variables (*p*s < 0.014).

### 3.2. Classification Performance of HCT for Hwabyung

**Figure 2:**
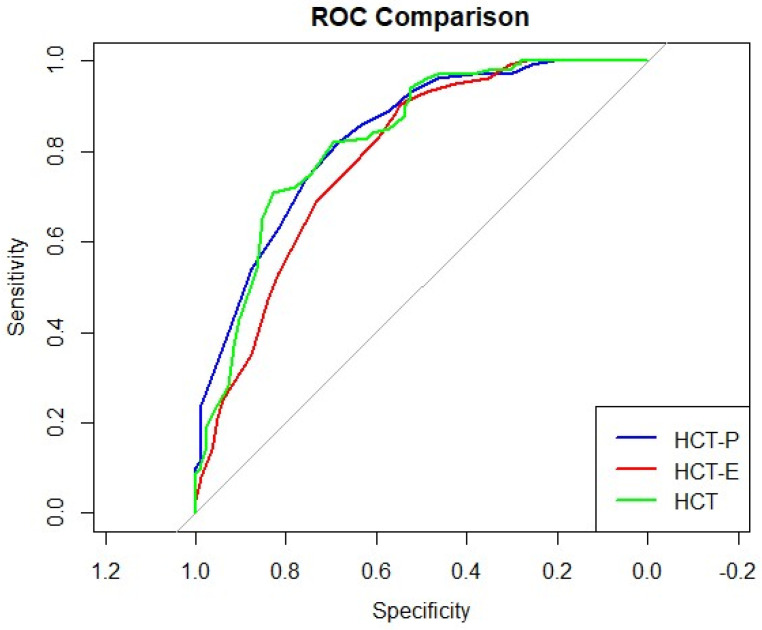
Receiver operating characteristic curves for identifying Hwabyung. AUC values: HCT = 0.826; HCT-P = 0.832; HCT-E = 0.785.

To evaluate the classification performance of the HCT, ROC curve analysis was conducted with HG as the positive group and NHG as the negative group (Figure 2). The AUC values were 0.826 (95% CI: 0.765–0.887) for HCT-total, 0.832 (95% CI: 0.773–0.890) for HCT-P, and 0.785 (95% CI: 0.717–0.853) for HCT-E. According to DeLong’s test, the AUC of HCT-total was significantly higher than that of HCT-E (*Z* = −3.054, *p* = 0.006; 95% CI for the AUC difference: −0.068 to −0.015) (Table 2).

The optimal cut-off score for HCT-total for identifying individuals with Hwabyung was 33.5 (95% CI: 22.5–34.5), yielding a sensitivity of 0.710 and a specificity of 0.829. Across the CI of the optimal cut-off, sensitivity ranged from 0.650 to 0.940, while specificity ranged from 0.524 to 0.854. As the cut-off decreases toward the lower bound, sensitivity increases while specificity decreases; conversely, as the cut-off increases toward the upper bound, sensitivity decreases and specificity increases. At the optimal cut-off, PPV was 0.835, NPV was 0.701, LR+ was 4.16, and LR− was 0.35 (Table 3).

For HCT-P, the optimal cut-off score was 13.5 (95% CI: 10.5–16.5), with a sensitivity of 0.820 and a specificity of 0.683. Across the CI of the optimal cut-off, sensitivity ranged from 0.540 to 0.930, while specificity ranged from 0.524 to 0.878. This pattern reflects the expected trade-off between sensitivity and specificity across cut-off values. The corresponding PPV was 0.759, NPV was 0.757, LR+ was 2.59, and LR− was 0.26 (Table 3).

### 3.3. Performance of the HCT in Discriminating Between Hwabyung and Other Psychiatric Conditions

**Figure 3:**
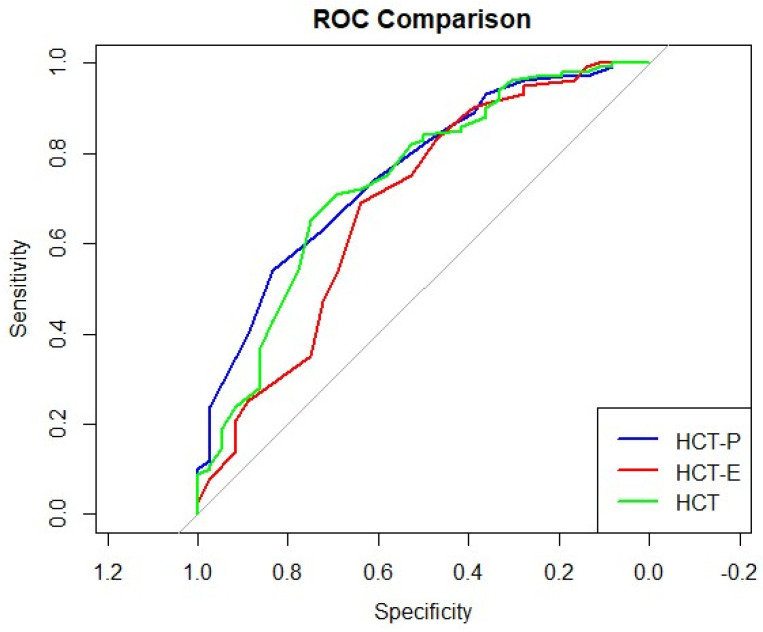
Receiver operating characteristic curves for distinguishing Hwabyung from other psychiatric conditions. AUC values: HCT = 0.737; HCT-P = 0.755; HCT-E = 0.685.

To evaluate the discriminative performance of the HCT for other psychiatric disorders, ROC curve analysis was conducted with HG as the positive group and NHCG as the negative group (Figure 3). The AUC values were 0.737 (95% CI: 0.638–0.835) for HCT-total, 0.755 (95% CI: 0.665–0.845) for HCT-P, and 0.685 (95% CI: 0.576–0.794) for HCT-E. According to DeLong’s test, the AUC of HCT-total was significantly higher than that of HCT-E (*Z* = −2.438, *p* = 0.045; 95% CI for the AUC difference: −0.094 to −0.010) (Table 2).

For differentiating Hwabyung from other psychiatric disorders, the optimal cut-off score for HCT-total was 33.5 (95% CI: 22.5–34.5), yielding a sensitivity of 0.710 and a specificity of 0.694. Across the CI of the optimal cut-off, sensitivity ranged from 0.650 to 0.940, while specificity ranged from 0.333 to 0.750. This pattern reflects the expected trade-off between sensitivity and specificity across cut-off values. At the optimal cut-off, PPV was 0.871, NPV was 0.460, LR+ was 2.32, and LR− was 0.42 (Table 3).

For HCT-P, the optimal cut-off score was 16.5 (95% CI: 10.5–17.5), with a sensitivity of 0.540 and a specificity of 0.833. Across the CI of the optimal cut-off, sensitivity ranged from 0.400 to 0.930, while specificity ranged from 0.361 to 0.889. This pattern reflects the expected trade-off between sensitivity and specificity across cut-off values. The corresponding PPV was 0.900, NPV was 0.395, LR+ was 3.24, and LR− was 0.55 (Table 3).

## 4. Discussion

This study examined the clinical validity of the HCT for identifying Hwabyung and for supporting its distinction from other psychiatric disorders. The findings indicate that the HCT demonstrates good validity for identifying Hwabyung and may provide useful probabilistic information when distinguishing Hwabyung from other psychiatric conditions.

HCT-total demonstrated stable and clinically meaningful performance across both screening and differential diagnostic decision-making contexts, supporting the use of a single cut-off score for initial identification of Hwabyung. The consistency of the optimal cut-off score suggests that HCT-total may serve as a pragmatic first-line screening tool applicable to heterogeneous hospital populations, including individuals with other psychiatric conditions as well as non-clinical visitors.

Although HCT-P demonstrated higher sensitivity and a lower LR− than HCT-total, indicating a relative advantage in minimizing false negatives, this gain in sensitivity was accompanied by lower specificity and a weaker capacity to increase post-test probability when results were positive. HCT-total, however, showed a more balanced performance profile, with higher specificity and greater overall discriminative stability. Given that Hwabyung is conceptualized as a psychosomatic condition encompassing both physical and emotional symptom domains [1,2], an exclusive focus on either physical or emotional symptoms alone may limit the clinical representation of the construct. In line with this, the relatively lower classification performance of HCT-E further underscores that, unlike depressive and anxiety disorders primarily linked to psychological distress across cognitive, emotional, and behavioral domains, Hwabyung is characterized by the co-occurrence of somatic symptoms such as chest discomfort and heat sensations alongside emotional distress; therefore, an integrative consideration of both domains is essential to capture its key pathological features. Accordingly, the selection of HCT-total for initial screening reflects a trade-off between maximizing sensitivity and maintaining conceptual comprehensiveness and interpretive balance within a heterogeneous clinical population.

In contrast, HCT-P demonstrated relatively higher specificity in distinguishing Hwabyung from other psychiatric disorders, along with higher PPV and a moderate LR+, suggesting that elevated HCT-P scores may increase the probability of Hwabyung in clinically ambiguous cases. However, given its limited sensitivity, HCT-P is best used as a complementary second-step measure following initial screening with HCT-total, rather than as a standalone diagnostic tool. This stepwise approach may enhance clinical decision-making by balancing sensitivity in screening with specificity in differentiation. HCT-P is therefore best conceptualized as a probabilistic indicator within a broader clinical assessment framework, rather than a definitive differential diagnostic test.

The inclusion of 95% CIs for the optimal cut-off scores provides additional insight into the clinical flexibility of the HCT. Rather than relying on a single fixed threshold, the observed range of plausible cut-off values illustrates how sensitivity and specificity shift across different decision points. At the lower bound of the CI, higher sensitivity and lower specificity suggest potential utility in contexts prioritizing the minimization of false negatives, such as initial screening in heterogeneous hospital populations. In contrast, at the upper bound, reduced sensitivity accompanied by increased specificity indicates greater suitability for contexts in which false positives carry greater clinical cost, such as differential evaluation among patients already presenting with psychiatric symptoms.

This pattern was consistently observed for both HCT-total and HCT-P. For example, HCT-total demonstrated high sensitivity at the lower bound and greater specificity at the upper bound, reflecting a trade-off between case detection and diagnostic precision. Similarly, HCT-P exhibited increases in specificity at higher thresholds, supporting its potential role in refining diagnostic impressions in more ambiguous clinical presentations. These findings suggest that the HCT may not need to be interpreted as a rigid dichotomous instrument; rather, it can be applied flexibly according to clinical priorities.

Taken together, these findings regarding diagnostic performance suggest that the HCT can be applied flexibly according to clinical priorities. HCT-total may be appropriate for initial case identification due to its broader construct coverage, whereas HCT-P may provide complementary value when greater specificity is desired in differential assessments. For example, lower cut-off thresholds may be preferred in screening contexts to maximize sensitivity, whereas higher thresholds may be applied in differential assessment settings where specificity is prioritized. However, such threshold selection should be guided by careful consideration of the trade-off between false-positive and false-negative classifications within a probabilistic clinical framework, rather than applied as a fixed diagnostic boundary.

The likelihood ratios observed in this study were in the small-to-moderate range, indicating only modest diagnostic value and a limited ability to substantially alter post-test probability. These findings should be taken into account when interpreting the clinical utility of the HCT. However, this pattern may reflect the nature of the HCT as a self-report instrument assessing subjective symptom patterns. While definitive diagnosis should be supported by comprehensive clinical evaluation, including clinician-administered interviews, the HCT may still provide clinically useful information for both screening and diagnostic decision-making.

The HG exhibited a higher proportion of females and married individuals compared with the NCG. This finding aligns with the traditional view of Hwabyung, which has been predominantly described among married middle-aged women experiencing chronic family-related stress, such as conflicts with spouses and in-laws [20,21]. However, recent studies suggest that Hwabyung is not limited to this demographic group and may also be prevalent among younger males, indicating the need for the development of updated conceptual models of Hwabyung [22]. Moreover, previous studies have reported that young men are more likely to experience problematic anger [23], and that anger- and violence-related behaviors have been identified as key mental health risks among adolescent males [24]. This indicates that anger-related problems in young men are becoming an increasingly significant public health concern.

The demographic distribution observed may partly reflect the characteristics of patients seeking care in Korean medicine hospitals, where middle-aged and older women constitute the majority of visitors. This pattern may, at least in part, be attributable to potential age and sex biases arising from convenience sampling. Such a demographic imbalance may limit the understanding of Hwabyung in younger adult men, and the cut-off values proposed for screening and differential support in this study may differ in this population. Therefore, caution is warranted when generalizing the present findings beyond the sampled population.

A high level of psychiatric comorbidity was observed in the Hwabyung group, with a substantial proportion of individuals presenting with at least one comorbid psychiatric disorder. These findings suggest the presence of shared transdiagnostic mechanisms underlying Hwabyung and other emotional disorders. In particular, difficulties in emotional regulation and a range of affective experiences, such as anger, sadness, and fear, may contribute to overlapping symptom presentations. Previous research has indicated that basic emotional states (e.g., anger, sadness, fear, disgust, and happiness) are often accompanied by similar bodily sensations, particularly in the chest region [6], supporting the possibility that Hwabyung shares common psychophysiological processes with other emotional disorders. From this perspective, Hwabyung may be understood not as a fully discrete diagnostic entity, but rather as a specific manifestation of broader emotional distress processes.

At the same time, Hwabyung is distinguished by the prominence of characteristic somatic symptoms, such as sensations of heat and increased pressure in the chest. These features are less emphasized in conventional mood and anxiety disorders and may contribute to its clinical distinctiveness. In this context, the HCT, which integrates both emotional and somatic symptom domains, may capture both shared and disorder-specific aspects of psychopathology. Notably, HCT-P demonstrated relatively higher specificity in differentiating Hwabyung from other psychiatric disorders, suggesting that the inclusion of somatic symptom dimensions may enhance discriminative validity.

Overall, these findings indicate that while the HCT may reflect both general psychopathology and Hwabyung-related features, its stepwise application—using HCT-total for initial assessment and HCT-P for further differentiation—may improve Hwabyung-specific classification. However, given the relatively small sample size for differential comparisons between Hwabyung and other psychiatric disorders, these findings should be considered exploratory, and further validation in larger and more diverse samples is warranted.

### Limitations

This study has some limitations. First, participants were recruited using a convenience sampling approach from a single hospital, which may introduce selection bias and limit the generalizability of the findings. This sampling method may have contributed to the demographic imbalance observed in the sample, particularly the underrepresentation of younger individuals and male participants. The overrepresentation of female participants in the present sample should be considered when interpreting the findings. Although the sample adequately reflects patients visiting a hospital, it did not sufficiently include younger individuals or male participants. Given that anger-related problems, such as interpersonal violence, have been reported more frequently among younger males [22,23,24], further research using more representative sampling strategies, such as stratified sampling, as well as studies focusing on underrepresented groups (e.g., younger male populations), is warranted. Such studies are necessary to better characterize Hwabyung across demographic groups and to develop a comprehensive Hwabyung model that accounts for both age and sex differences.

Second, this study was conducted in a single country, reflecting the East Asian cultural context. Although Hwabyung is often regarded as a culture-bound syndrome specific to Korea, anger is recognized as a basic human emotion, and its association with interoceptive processes is supported by broadly accepted empirical evidence [25,26]. From a broader perspective, Hwabyung may be understood in relation to transdiagnostic constructs of emotional distress, particularly those involving emotion dysregulation and shared psychophysiological responses. Therefore, future research should examine the clinical identification, assessment, and monitoring of Hwabyung across diverse cultural contexts. To establish the international validity of the HCT, repeated validation studies examining its screening and differential diagnostic performance in different cultural settings are needed.

Third, this study is limited by the relatively small sample size of the NHCG. This limitation restricts the precision of estimates for differential performance between Hwabyung and other psychiatric conditions. Consequently, the findings regarding the discriminative ability of the HCT should be interpreted as preliminary. Future studies should seek to replicate and extend these findings using larger, multi-center samples that include a broader range of psychiatric diagnoses, thereby enabling more robust estimation of classification accuracy and improving the generalizability of the results.

## 5. Conclusions

HCT-total and HCT-P demonstrated promising classification performance for identifying individuals with Hwabyung and fair discriminative performance for differentiating Hwabyung from other psychiatric disorders. For primary screening, a cut-off score of 33.5 for HCT-total may be useful for capturing the overall clinical presentation of Hwabyung. In addition, a cut-off score of 16.5 for HCT-P may serve as a supplementary measure to enhance specificity in the context of differentiating Hwabyung from other psychiatric conditions.

These findings should be interpreted within a probabilistic framework, taking into account the trade-off between sensitivity and specificity across different threshold values. In particular, caution is warranted in interpreting these results given the potential influence of sampling bias and the relatively small sample size for differential comparisons with other psychiatric disorders. Further validation in larger, more diverse, and representative clinical samples is warranted to establish the robustness and generalizability of the HCT.

## Figures and Tables

**Table 1:** Demographic and clinical characteristics (mean and standard deviation) of the Hwabyung, non-Hwabyung clinical, and non-clinical groups.

Measure	Mean ± SD or Rate% (*n*)			*F*/*χ*^2^	*p*	Post Hoc Analysis
	HG (*n* = 100; A)	NHCG (*n* = 36; B)	NCG (*n* = 46; C)			
Age	53.74 ± 7.91	54.86 ± 9.60	50.52 ± 11.89	2.597	0.077	A = B = C
Gender						
Male	3.00 (3)	8.3 (3)	15.2 (7)	7.186	0.028	A > C ^a^
Female	97.00 (97)	91.7 (33)	84.8 (39)			
Marital status						
Unmarried	5.00 (5)	5.6 (2)	17.4 (8)	6.825	0.033	A > C ^b^
Married	95.00 (95)	94.4 (34)	82.6 (38)			
HCT	35.10 ± 7.07	27.00 ± 10.70	17.67 ± 11.85	57.226	<0.001	A > B > C
HCT-P	16.22 ± 3.59	12.17 ± 4.84	7.89 ± 5.45	58.557	<0.001	A > B > C
HCT-E	18.88 ± 4.23	14.83 ± 6.42	9.78 ± 6.67	45.491	<0.001	A > B > C
BDI	23.32 ± 8.79	18.67 ± 8.22	10.87 ± 6.61	34.848	<0.001	A > B > C
BAI	24.23 ± 11.87	17.39 ± 10.55	8.37 ± 6.65	36.209	<0.001	A > B > C

Note. ^a^ = Proportion of females compared with males. ^b^ = Proportion of married participants compared with unmarried participants. BAI = Korean version of Beck Anxiety Inventory; BDI = Korean version of Beck Depression Inventory; HCT = Hwabyung Comprehensive Test, symptom scale (total score); HCT-E = emotional symptom subscale of the HCT; HCT-P = physical symptom subscale of the HCT; HG = Hwabyung group; NCG = non-clinical group; NHCG = non-Hwabyung clinical group.

**Table 2:** Pairwise comparisons of AUC values using DeLong’s test.

Classification Task	Comparison	*Δ*AUC	*Z*	*p* ^a^	95% CI for *Δ*AUC
HG vs. NHG	HCT vs. HCT-P	−0.005	0.355	1.000	−0.024 to 0.035
	HCT vs. HCT-E	0.041	−3.054	0.006	−0.068 to −0.015
	HCT-P vs. HCT-E	0.047	1.789	0.222	−0.004 to 0.098
HG vs. NHCG	HCT vs. HCT-P	−0.018	0.660	1.000	−0.036 to 0.073
	HCT vs. HCT-E	0.052	−2.438	0.045	−0.094 to −0.010
	HCT-P vs. HCT-E	0.070	1.586	0.339	−0.017 to 0.157

Note. ^a^ Bonferroni correction was applied within each classification task for the three pairwise AUC comparisons. AUC = area under the curve; HCT = Hwabyung Comprehensive Test, symptom scale (total score); HCT-E = emotional symptom subscale of the HCT; HCT-P = physical symptom subscale of the HCT; HG = Hwabyung group; NHCG = non-Hwabyung clinical group; NHG = non-Hwabyung group.

**Table 3:** Cross-tabulation of HCT results and clinical diagnosis of Hwabyung.

Classification Task	Index Test		Reference Standard		
			Positive Case	Negative Case	Total
HG vs. NHG	HCT	≥33.5 points	71 (71.0)	14 (17.1)	85 (46.7)
		<33.5 points	29 (29.0)	68 (82.9)	97 (53.3)
		Total	100 (54.9)	82 (45.1)	182 (100)
	HCT-P	≥13.5 points	82 (82.0)	26 (31.7)	108 (59.3)
		<13.5 points	18 (18.0)	56 (68.3)	74 (40.6)
		Total	100 (54.9)	82 (45.1)	182 (100)
	HCT-E	≥13.5 points	90 (90.0)	37 (45.1)	127 (69.8)
		<13.5 points	10 (10.0)	45 (54.9)	55 (30.2)
		Total	100 (54.9)	82 (45.1)	182 (100)
HG vs. NHCG	HCT	≥33.5 points	71 (71.0)	11 (30.6)	82 (60.3)
		<33.5 points	29 (29.0)	25 (69.4)	54 (39.7)
		Total	100 (73.5)	36 (26.5)	136 (100)
	HCT-P	≥16.5 points	54 (54.0)	6 (16.7)	60 (44.1)
		<16.5 points	46 (46.0)	30 (83.3)	76 (55.9)
		Total	100 (73.5)	36 (26.5)	136 (100)
	HCT-E	≥17.5 points	69 (69.0)	13 (36.1)	82 (60.3)
		<17.5 points	31 (31.0)	23 (63.9)	54 (39.7)
		Total	100 (73.5)	36 (26.5)	136 (100)

Note. HCT = Hwabyung Comprehensive Test, symptom scale (total score); HCT-E = emotional symptom subscale of the HCT; HCT-P = physical symptom subscale of the HCT; HG = Hwabyung group; NHCG = non-Hwabyung clinical group; NHG = non-Hwabyung group. Values are presented as number (%).

## Data Availability

The datasets used and/or analyzed during the current study are available from the corresponding author upon reasonable request.

## Supplementary Materials

The following supporting information can be downloaded at: https://www.mdpi.com/article/10.3390/diagnostics16081165/s1, Supplementary Material S1: STARD checklist.

## Abbreviations

The following abbreviations are used in this manuscript:

HS	Hwabyung Scale
HCT	Hwabyung Comprehensive Test
HCT-P	Hwabyung Comprehensive Test physical symptoms subscale
HCT-E	Hwabyung Comprehensive Test emotional symptoms subscale
ROC	receiver operating characteristic
AUC	area under the curve
BDI	Korean version of the Beck Depression Inventory
BAI	Korean version of the Beck Anxiety Inventory
HG	Hwabyung group
NHG	non-Hwabyung group
NHCG	non-Hwabyung clinical group
NCG	non-clinical group
CI	confidence interval
PPV	positive predictive value
NPV	negative predictive value
LR+	positive likelihood ratio
LR−	negative likelihood ratio
